# Evaluation of outcome of chemotherapy for breast cancer patients older than 70 years: A SEER-based study

**DOI:** 10.3389/fonc.2023.992573

**Published:** 2023-03-28

**Authors:** Shengyu Pu, Peiling Xie, Heyan Chen, Yijun Li, Jianjun He, Huimin Zhang

**Affiliations:** Department of Breast Surgery, the First Affiliated Hospital of Xi’an Jiaotong University, Xi’an, Shaan’xi, China

**Keywords:** elderly breast cancer, chemotherapy, nomogram, inverse probability of treatment weighting (IPTW), breast cancer-specific survival (BCSS)

## Abstract

**Background:**

With the aging of the population, the number of elderly breast cancer cases has increased. However, there is a lack of effective randomized clinical trial data to support whether elderly patients should receive chemotherapy. Our goal was to observe the relationship between chemotherapy and breast cancer-specific survival (BCSS) in elderly breast cancer patients and to identify those who could benefit from chemotherapy.

**Methods:**

We collected the data of patients who were diagnosed with invasive ductal carcinoma and older than 70 years in the SEER database from 1995 to 2016. The independent predictors of BCSS were identified by Cox regression analysis. Propensity score matching (PSM) and inverse probability of treatment weighting (IPTW) were performed to eliminate confounding factors.

**Results:**

A total of 142,537 patients were collected, including 21,782 patients in the chemotherapy group and 120,755 patients in the non-chemotherapy group. We identified the same potential predictors of BCSS after PSM and IPTW, such as age, race, grade, stage, therapy, subtype. A nomogram for predicting 3-year, 5-year and 10-year BCSS was constructed. The 3-year, 5-year and 10-year AUCs of the nomogram were 0.842, 0.819, and 0.788. According to the risk stratification of model predictive scores, patients in the high-risk group achieved the greatest improvement in BCSS after receiving chemotherapy.

**Conclusions:**

Our study suggests that women older than 70 years with larger tumors, higher grade, positive nodes, negative hormone receptor and inactive local therapy gain prognostic benefits from chemotherapy, but for those with low- and median-risk, conventional chemotherapy should be administered cautiously.

## 1 Introduction

Breast cancer is the malignant tumor with the highest incidence in the world and the highest mortality rate among women, and the number of breast cancer-related deaths ranks fifth among all tumor types (1). With the aging of the population, the number of elderly breast cancer cases has increased (2). The pathological characteristics of elderly breast cancer patients are relatively indolent, but the tumor mostly has a higher stage and distant metastasis when diagnosed (3). In addition, due to more comorbidities and poor treatment tolerance, elderly patients usually received less adjuvant therapy, resulting in higher breast cancer-specific mortality. Current studies showed that women >=70 years old accounted for 31% of all breast cancer cases, but they constituted 47% of all breast cancer-specific deaths (4). On the other hand, due to the under-representation of elderly cancer patients in clinical trials, there is insufficient research evidence on whether elderly patients receive chemotherapy. In clinical trials conducted by the National Cancer Institute of the United States, only a small number of elderly patients was included, with 25% were 65-74 years old and 10% were >=75 years old (5). A prospective trial suggested that elderly patients who are in good general health could benefit from adjuvant chemotherapy in a similar way to younger women (6). Overall, despite the increased incidence of breast cancer in elderly patients, there is little evidence to help doctors decide whether chemotherapy is required for patients over the age of 70.

In this study, we extracted data from the SEER (Surveillance, Epidemiology, and End Results) database to explore the breast cancer-specific survival (BCSS) of elderly breast cancer patients. Then, a predictive model was established and verified to screen out those who could benefit from chemotherapy and provide a reference for clinical decision making.

## 2 Materials and methods

### 2.1 Study population

This is a retrospective cohort study with data from the SEER database. The SEER database is a large tumor database covering approximately 34.6% of the U.S. population. The data were derived from case data of patients with malignant tumors in 18 states representing all regions of the United States, including detailed data on morbidity, mortality, and basic treatment methods (7). All data were downloaded *via* SEER*Stat software, and procedures were performed in accordance with approved guidelines. Since the SEER database is publicly accessible, informed patient consent was not required for this study.

### 2.2 Data collection

**Figure 1:**
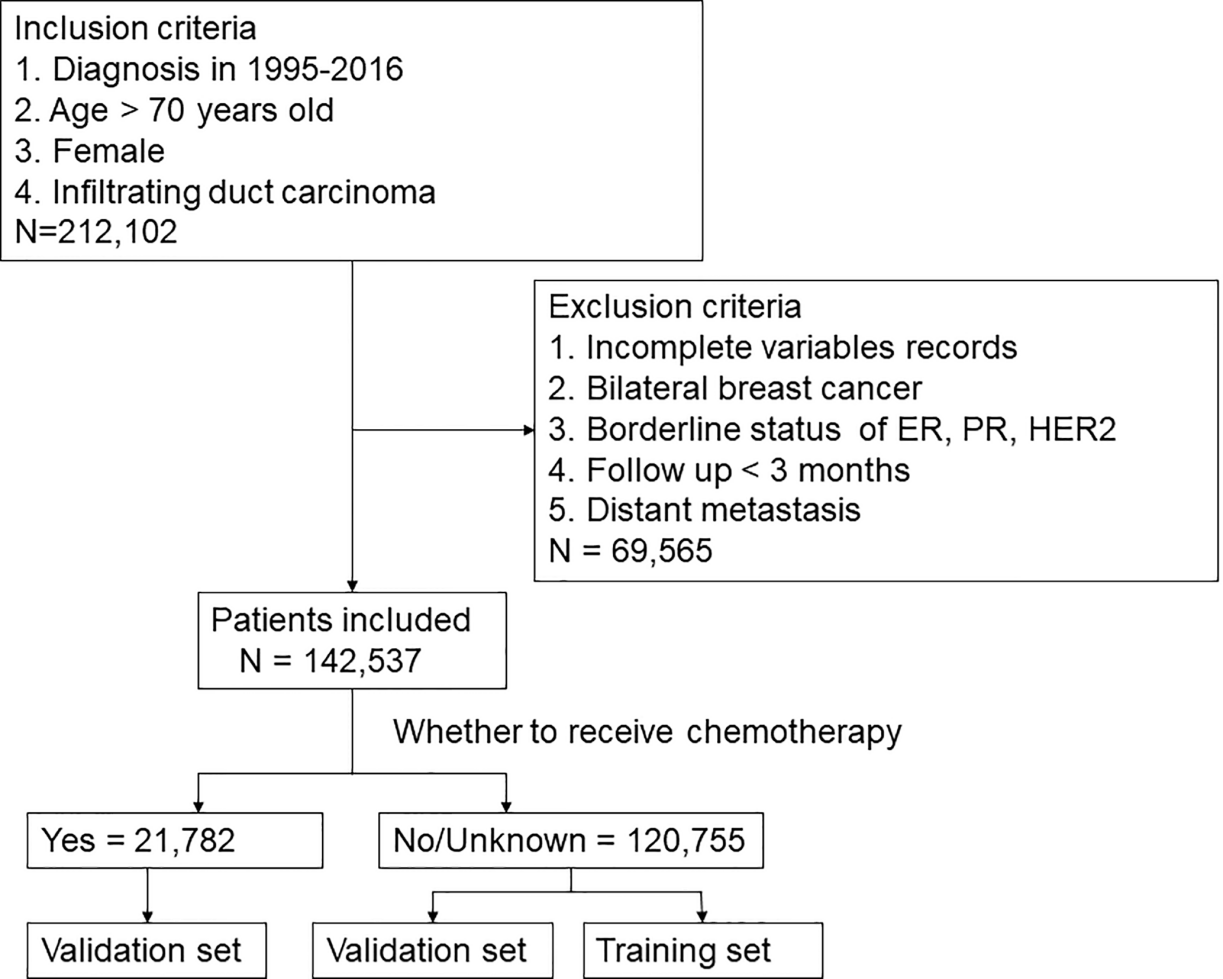
Flow Diagram of selection method.

This study collected the data of all patients who were diagnosed with invasive ductal carcinoma (ICD-0-3 histology codes: 8500/3) and over 70 years old in the SEER database from 1995 to 2016. Demographic characteristics included age, marital status, and race. Age was analyzed as a categorical variable and classified into three groups by X-tile (8): 70-77 years old, 78-84 years old, and >=85 years old. Breast cancer-related characteristics, such as laterality of the tumor, grade, TNM stage, ER and PR status, HER2 status, radiation, chemotherapy, surgery, duration of follow-up and survival status, were collected. Data with the following characteristics were excluded (1): <70 years old; (2) male breast cancer; (3) duration of follow-up <3 months; (4) distant metastasis; (5) missing data; and (6) bilateral breast cancer. Ultimately, 142,537 patients were included in this study. The selection procedure was shown in **Figure 1**. The endpoint was BCSS, which was calculated from the date of diagnosis to the date of death from breast cancer.

### 2.3 Statistical analysis

The included patients were divided into two groups according to whether they received chemotherapy. For adjusting between-group differences, the propensity scores were developed with the use of multivariate Logistic regression based on the following characteristics: age, race, marital status, laterality, grade, AJCC 6^th^ stage, T stage, N stage, local therapy and subtype. We used propensity score matching (PSM) and inverse probability of treatment weighting (IPTW) to eliminate confounding factors (9). The patients who received chemotherapy were matched 1:1 to patients who did not receive chemotherapy on propensity score with a greedy matching algorithm (a caliper width of 0.2 of the pooled standard deviation). For IPTW, we applied the inverse propensity score as weights for patients who received chemotherapy and the inverse of 1 minus the propensity score for patients who did not. The standardized mean difference (SMD) was used to assess the difference in distribution between groups for each variable after matching and weighting. A SMD<10% means that there is no significant difference. The unmatched data were analyzed using the Pearson chi-square test.

After adjusting the data with PSM and IPTW, we divided the patients into training set and validation set (7:3) in the non-chemotherapy group. In the training set, a nomogram for predicting BCSS at 3 years, 5 years, and 10 years was established according to the significant factors screened in the Cox univariate and multivariate analyses. The discrimination and correction of the nomogram were evaluated both in the validation group and chemotherapy group. The discrimination was assessed using the time-dependent area under the ROC curve (AUC), which ranges from 0.5 to 1.0. A value of 1 indicates that the model can predict 100% without errors, and 0.5 indicates that the model has no predictive ability. The larger the AUC within this range, the higher the diagnostic accuracy of the model. The calibration curve was used to evaluate the correction of the model. When the curve is highly coincident with the diagonal, the calibration of the model is optimal. Moreover, we conducted the Decision Curve Analysis (DCA) to observe the clinical utility of the model. Finally, X-tile software program selected the best cutoff of predictive scores in the model by the highest X^2^ value, and calculated the minimum *P* value by the log-rank test. The entire cohort was divided into high-, median- and low-risk groups. Kaplan–Meier curve analysis was employed to generate BCSS curves, and the log-rank test was performed to determine the significant difference among groups.

All statistical analyses were conducted with X-tile (3.6.1, Yale University 2003-2005), IBM SPSS Statistics 24.0 software (IBM Corporation, Armonk, NY, USA) and R statistical software (version 4.1.1, R Foundation for Statistical Computing, Vienna, Austria). A two-tailed P<0.05 was considered statistically significant.

## 3 Results

### 3.1 The clinicopathological characteristics of the study population

**Figure 2:**
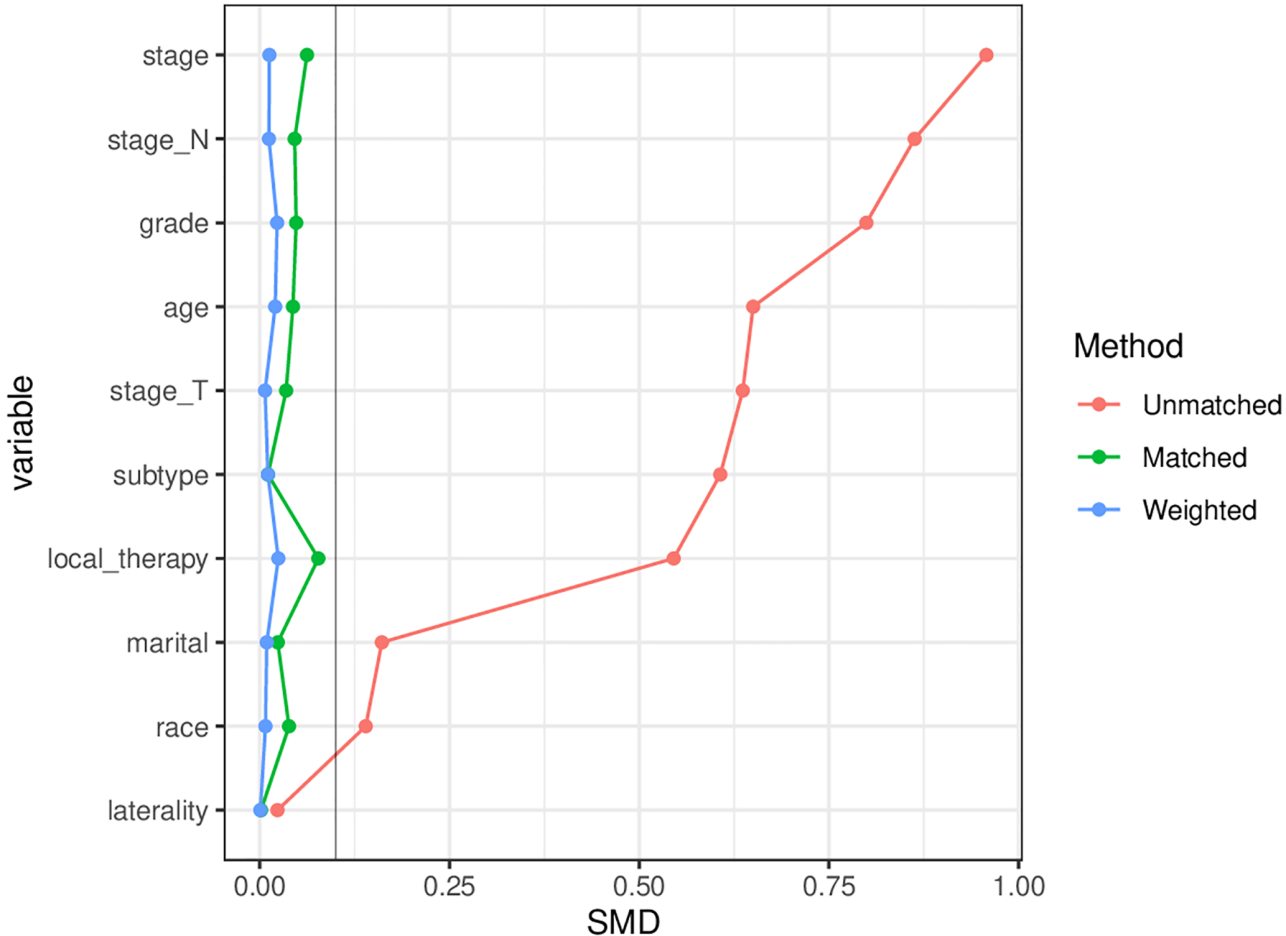
Comparison of the matching effects of the propensity score matching (PSM) and inverse probability of treatment weighting (IPTW). SMD, Standardized mean difference, A SMD<10% means that there is no significant difference between the distribution of variables.

In this study, 142,537 elderly breast cancer patients were collected from the SEER database, including 21,782 patients in the chemotherapy group and 120,755 patients in the non-chemotherapy group. The median duration of follow-up was 62 months. **Table 1** showed the specific clinicopathological characteristics, including age, race, marital status, grade, laterality, T stage, N stage, local therapy, and molecular subtype. The chi-square test showed that the distribution of each variable in the two groups before matching was unbalanced. After PSM (**Supplementary Table 1**) and IPTW, a sufficient balance of all covariates was achieved between the chemotherapy group and the non-chemotherapy group. According to **Figure 2**, the matching effect of IPTW was more adequate. Among the overall patients, patients who were 70-77 years old and white accounted for the majority. In the local therapy, most of the patients received breast-conserving surgery combined with radiation. Among elderly breast cancer patients, the most common subtype was HR+/HER2-, except for patients whose HER2 status were unknown.

**Table 1:** The clinicopathological characteristics of the unmatched and inverse probability of treatment weighting (IPTW) matched patients.

	Unmatched			IPTW Matched		
Variables	No/Unknown	Yes		No/Unknown	Yes	
	N=120755 (%)	N=21782 (%)	*P*-value	N=17544.9 (%)	N=17747.9 (%)	*SMD*
Age						
70-77	60443 (50.1)	17019 (78.1)	0.000	13008.3 (74.1)	13307.9 (75.0)	0.020
78-84	40587 (33.6)	4077 (18.7)		3828.1 (21.8)	3761.4 (21.2)	
>=85	19725 (16.3)	686 (3.15)		708.6 (4.0)	678.5 (3.8)	
Race						
Black	8342 (6.91)	2333 (10.7)	0.000	1812.5 (10.3)	1793.6 (10.1)	0.007
Other	7153 (5.92)	1411 (6.48)		1110.4 (6.3)	1130.8 (6.4)	
White	105260 (87.2)	18038 (82.8)		14622.0 (83.3)	14823.4 (83.5)	
Marital						
No	71242 (59.0)	11067 (50.8)	0.000	9199.8 (52.4)	9223.7 (52.0)	0.009
Yes	49513 (41.0)	10715 (49.2)		8345.1 (47.6)	8524.2 (48.0)	
Laterality						
Left	61207 (50.7)	11297 (51.9)	0.001	9036.3 (51.5)	9149.6 (51.6)	0.001
Right	59548 (49.3)	10485 (48.1)		8508.6 (48.5)	8598.3 (48.4)	
Grade						
I	32655 (27.0)	1577 (7.24)	0.000	1474.6 (8.4)	1551.5 (8.7)	0.022
II	57871 (47.9)	7393 (33.9)		6540.2 (37.3)	6746.8 (38.0)	
III	29494 (24.4)	12576 (57.7)		9340.1 (53.2)	9260.9 (52.2)	
IV	735 (0.61)	236 (1.08)		190.0 (1.1)	188.6 (1.1)	
Stage						
I	78634 (65.1)	5328 (24.5)	0.000	5107.3 (29.1)	5268.3 (29.7)	0.013
II	34917 (28.9)	10586 (48.6)		8897.0 (50.7)	8914.1 (50.2)	
III	7204 (5.97)	5868 (26.9)		3540.6 (20.2)	3565.4 (20.1)	
T stage						
T1	88328 (73.1)	9572 (43.9)	0.000	8523.6 (48.6)	8677.1 (48.9)	0.008
T2	27328 (22.6)	9346 (42.9)		7177.8 (40.9)	7237.0 (40.8)	
T3	2607 (2.16)	1362 (6.25)		908.9 (5.2)	896.6 (5.1)	
T4	2492 (2.06)	1502 (6.90)		934.6 (5.3)	937.1 (5.3)	
N stage						
N0	98727 (81.8)	9663 (44.4)	0.000	8834.4 (50.4)	9028.5 (50.9)	0.011
N1	17296 (14.3)	7584 (34.8)		6047.1 (34.5)	6029.6 (34.0)	
N2	3256 (2.70)	2907 (13.3)		1789.1 (10.2)	1811.3 (10.2)	
N3	1476 (1.22)	1628 (7.47)		874.3 (5.0)	878.5 (4.9)	
Local_therapy						
BCS	26206 (21.7)	2569 (11.8)	0.000	2232.6 (12.7)	2283.8 (12.9)	0.025
BCS+Radiation	46644 (38.6)	7629 (35.0)		6400.9 (36.5)	6657.3 (37.5)	
Mastectomy	40675 (33.7)	7108 (32.6)		6397.8 (36.5)	6292.1 (35.5)	
Mastectomy+Radiation	3414 (2.83)	3737 (17.2)		1949.5 (11.1)	1963.4 (11.1)	
Radiation	32 (0.03)	31 (0.14)		14.3 (0.1)	13.7 (0.1)	
No	3784 (3.13)	708 (3.25)		549.8 (3.1)	537.5 (3.0)	
Subtype						
HR-/HER2-	3399 (2.81)	2542 (11.7)	0.000	1610.9 (9.2)	1628.3 (9.2)	0.009
HR-/HER2+	957 (0.79)	1114 (5.11)		582.4 (3.3)	582.6 (3.3)	
HR+HER2-	40380 (33.4)	4194 (19.3)		3739.0 (21.3)	3848.5 (21.7)	
HR+/HER2+	2717 (2.25)	2103 (9.65)		1300.3 (7.4)	1318.2 (7.4)	
Not 2010+	73302 (60.7)	11829 (54.3)		10312.3 (58.8)	10370.1 (58.4)	

BCS, Breast conserving surgery; HR, Hormone receptor; HER2, Human epidermal growth factor receptor 2; IPTW, Inverse probability of treatment weighting; SMD, Standardized mean differences.

### 3.2 Predictors of BCSS for non-chemotherapy patients

Combined with clinical and statistical significance, we screened out the same potential predictors of BCSS after PSM (**Supplement Table 2**) and IPTW (**Table 2**), which included age, race, grade, T stage, N stage, local therapy and subtype. Being younger than 85 years old (such as 70-77 vs >=85 years old, HR=0.507, 95% CI: 0.422-0.609) and being white (white vs black, HR=0.819, 95% CI: 0.709-0.947) were protective factors for BCSS. The patients with higher grade (*P*=0.000), T stage (*P*=0.000), and N stage (*P*=0.000) had a worse prognosis. Regarding molecular subtype, HR-HER2- had a worse prognosis than other subtypes (such as HR+HER2+ vs HR-HER2-, HR=0.528, 95% CI: 0.447-0.623). Compared with breast-conserving surgery (BCS) alone, BCS combined with radiation improved outcome (HR=0.631, 95% CI: 0.538-0.742), and radiation alone (HR=2.270, 95% CI: 1.187-4.342) was associated with worse outcome. Marital status and laterality were not statistically associated with BCSS.

**Table 2:** Univariate and multivariable analysis of breast cancer-specific survival (BCSS) predictors in patients after inverse probability of treatment weighting (IPTW).

Variables	Univariate analysis			Multivariate analysis		
	HR*	95%CI	*P-*value	HR*	95%CI	*P-*value
Age						
>=85	Reference			Reference		
70-77	0.459	(0.373-0.564)	0.000	0.507	(0.422-0.609)	0.000
78-84	0.618	(0.498-0.768)	0.000	0.692	(0.570-0.842)	0.000
Race						
Black	Reference			Reference		
Other	0.492	(0.370-0.653)	0.000	0.625	(0.477-0.818)	0.001
White	0.601	(0.522-0.692)	0.000	0.819	(0.709-0.947)	0.007
Laterality						
Left	Reference					
Right	0.916	(0.826-1.015)	0.094			
Marital						
No	Reference			Reference		
Yes	0.724	(0.657-0.797)	0.000	0.931	(0.846-1.024)	0.141
Grade						
I	Reference			Reference		
II	1.915	(1.512-2.424)	0.000	1.451	(1.131-1.860)	0.003
III	3.802	(3.065-4.716)	0.000	2.263	(1.812-2.828)	0.000
IV	4.34	(3.193-5.901)	0.000	2.832	(2.069-3.878)	0.000
T stage						
T1	Reference			Reference		
T2	2.91	(2.588-3.272)	0.000	1.739	(1.533-1.973)	0.000
T3	6.748	(5.645-8.066)	0.000	2.586	(2.093-3.197)	0.000
T4	8.542	(7.421-9.833)	0.000	2.687	(2.280-3.165)	0.000
N stage						
N0	Reference			Reference		
N1	2.046	(1.848-2.266)	0.000	1.516	(1.386-1.657)	0.000
N2	4.606	(4.120-5.150)	0.000	2.630	(2.372-2.916)	0.000
N3	8.271	(7.335-9.327)	0.000	3.779	(3.363-4.246)	0.000
Local_therapy						
BCS	Reference			Reference		
BCS+Radiation	0.561	(0.477-0.660)	0.000	0.631	(0.538-0.742)	0.000
Mastectomy	1.456	(1.239-1.711)	0.000	1.136	(0.951-1.356)	0.160
Mastectomy+Radiation	2.846	(2.444-3.314)	0.000	1.015	(0.850-1.213)	0.867
Radiation	6.079	(3.187-1.594)	0.000	2.270	(1.187-4.342)	0.013
No	6.292	(4.879-8.116)	0.000	3.714	(2.850-4.839)	0.000
Subtype						
HR-/HER2-	Reference			Reference		
HR-/HER2+	0.961	(0.783-1.180)	0.705	0.786	(0.643-0.962)	0.019
HR+/HER2-	0.351	(0.278-0.443)	0.000	0.562	(0.454-0.696)	0.000
HR+/HER2+	0.533	(0.448-0.635)	0.000	0.528	(0.447-0.623)	0.000
Not 2010+	0.424	(0.379-0.475)	0.000	0.620	(0.556-0.692)	0.000

BCS, Breast conserving surgery; HR, Hormone receptor; HER2, Human epidermal growth factor receptor 2; HR*, hazard ratio; CI, confidence interval.

### 3.3 Construction and validation of the nomogram for BCSS

**Figure 3:**
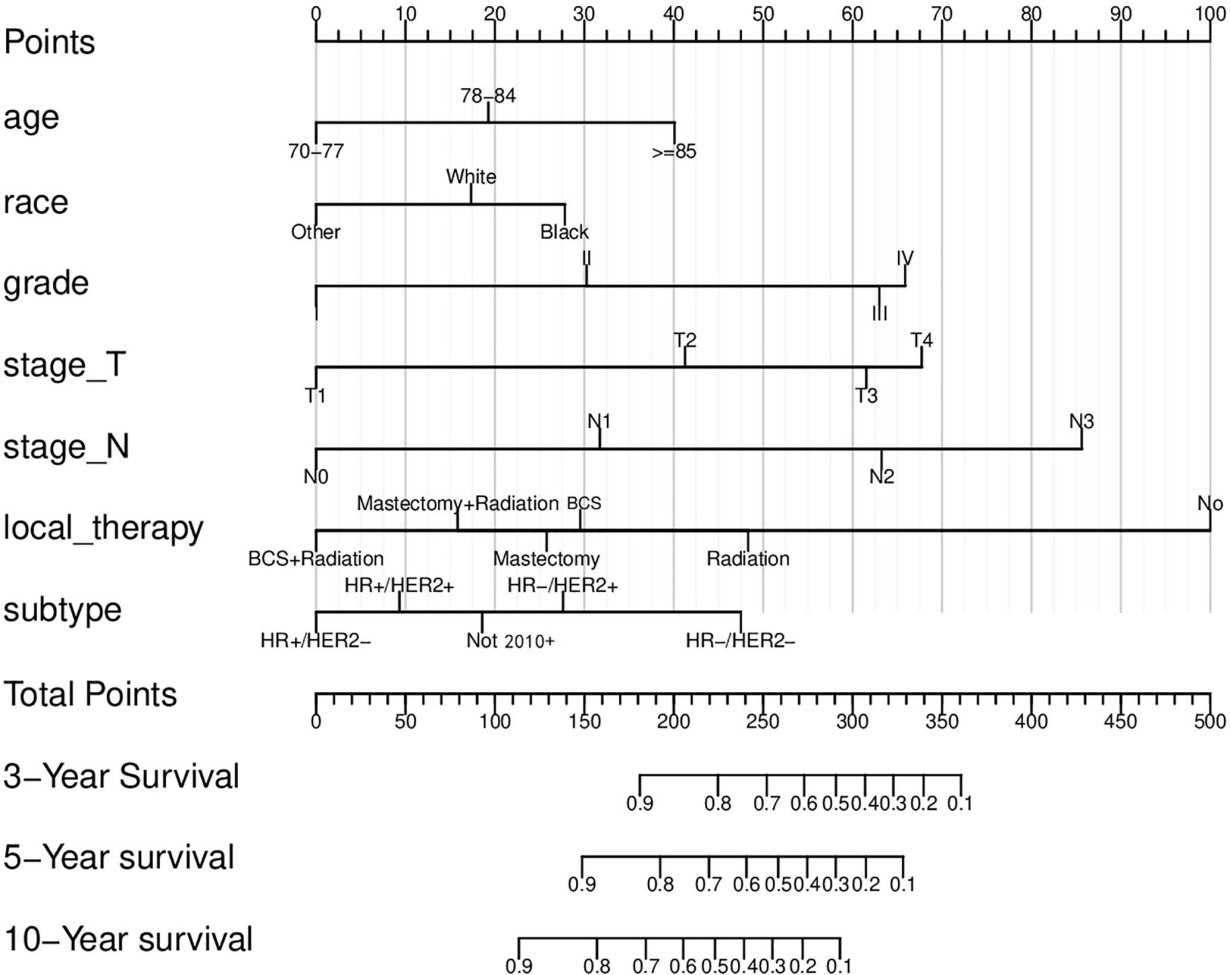
The nomogram to predict the breast cancer-specific survival (BCSS) rates. BCS, Breast conserving surgery; HR, Hormone receptor; HER2, Human epidermal growth factor receptor 2.

**Figure 4:**
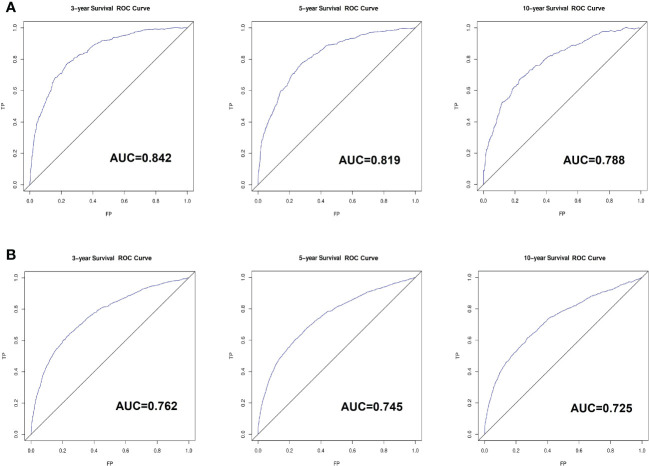
The receiver-operating characteristics (ROC) curve for validating nomogram in the non-chemotherapy group **(A)** and chemotherapy group **(B)**. TP, True Positive; FP, False Positive.

**Figure 5:**
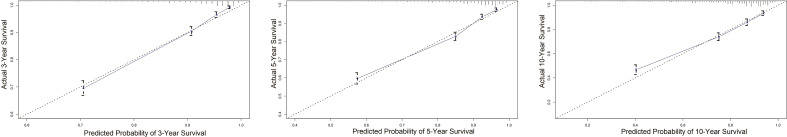
The calibration plot for validating nomogram in the non-chemotherapy group. When the curve is highly coincident with the diagonal, the predicted probability of survival is highly consistent with the actual survival, which means the model has excellent predictive performance.

**Figure 6:**
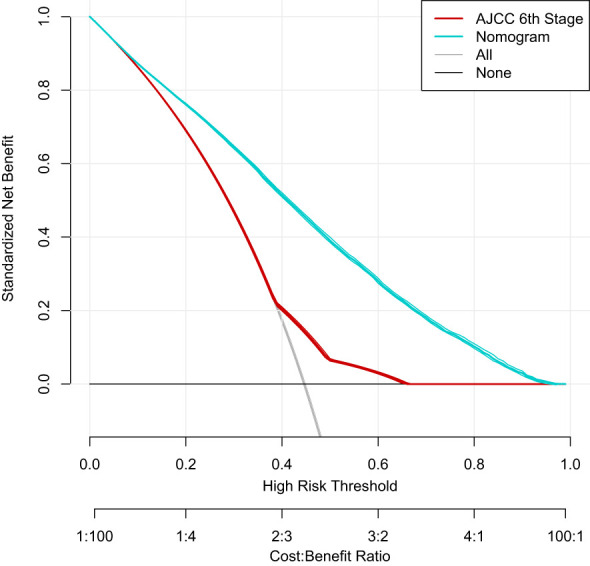
The Decision Curve Analysis (DCA) curve for the nomogram and AJCC 6^th^ stage.

Based on the factors screened out by univariate and multivariate Cox regression analysis, a nomogram for predicting 3-year, 5-year and 10-year BCSS was constructed, as shown in **Figure 3**. According to the risk score of each variable (**Table 3**), a patient’s total score can be calculated, and then the corresponding 3-year, 5-year and 10-year BCSS can be estimated from the nomogram. The predictive performance of the nomogram was evaluated both in the validation group and chemotherapy group. The ROC curve of the nomogram was shown in **Figure 4**; the 3-year, 5-year and 10-year AUCs in the validation group (A) were 0.842, 0.819, and 0.788, while those in the chemotherapy group (B) were 0.762, 0.745, and 0.725, respectively. The calibration chart showed good agreement between the predicted probability and the observed probability (**Figures 5**; **S1**). As shown in the DCA curve (**Figure 6**), the clinical utility of nomogram was better than AJCC 6^th^ stage.

**Table 3:** The risk score to predict breast cancer-specific survival (BCSS) rates according to nomogram.

	Variables	Points
**Age**	>=85	40
	70-77	0
	78-84	19
**Race**	Black	28
	Other	0
	White	17
**Grade**	I	0
	II	30
	III	63
	IV	66
**T stage**	T1	0
	T2	41
	T3	62
	T4	68
**N stage**	N0	0
	N1	32
	N2	63
	N3	86
**Local_therapy**	BCS	30
	BCS+Radiation	0
	Mastectomy	26
	Mastectomy+Radiation	16
	No	100
	Radiation	48
**Subtype**	HR-/HER2-	47
	HR-/HER2+	28
	HR+/HER2-	0
	HR+/HER2+	9
	Not 2010+	19

BCS, Breast conserving surgery; HR, Hormone receptor; HER2, Human epidermal growth factor receptor 2.

### 3.4 Influence of chemotherapy on the BCSS of specific risk stratification

**Figure 7:**
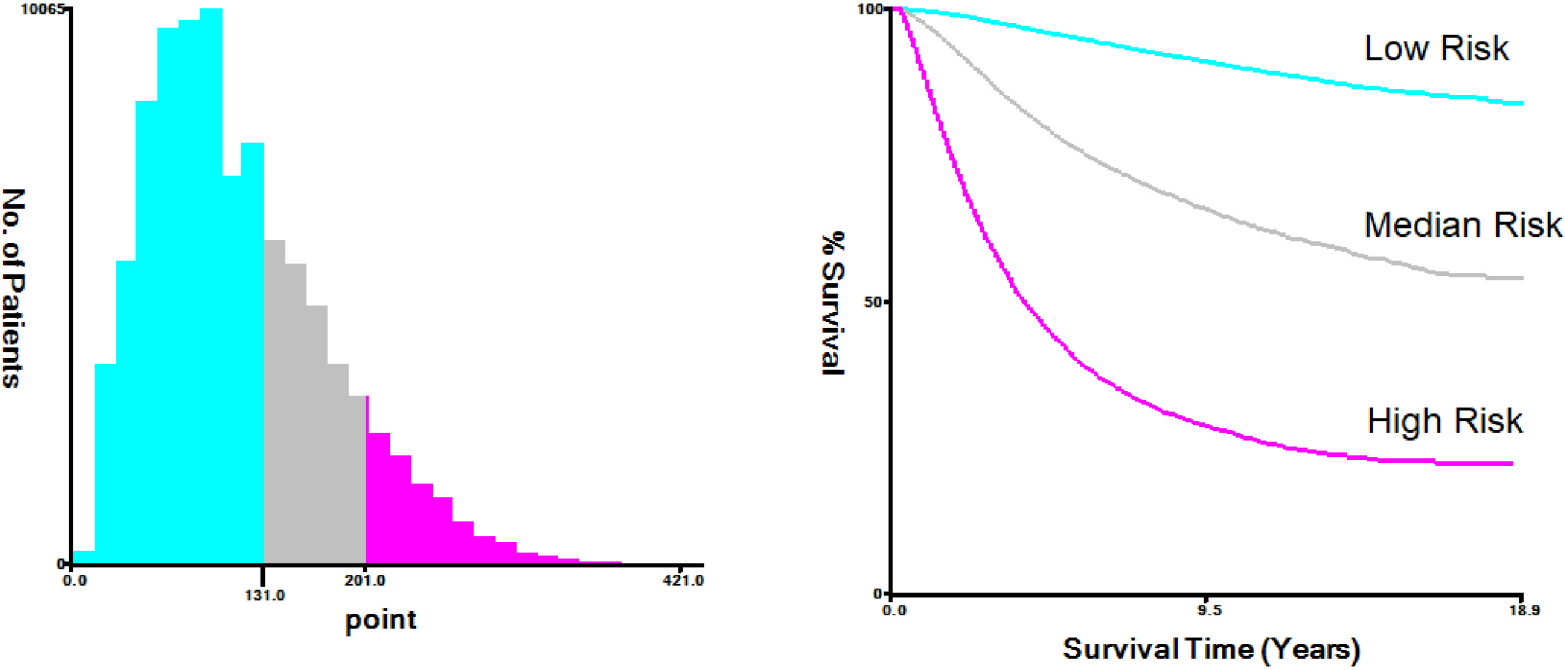
X-tile analysis of the risk stratification.

**Figure 8:**
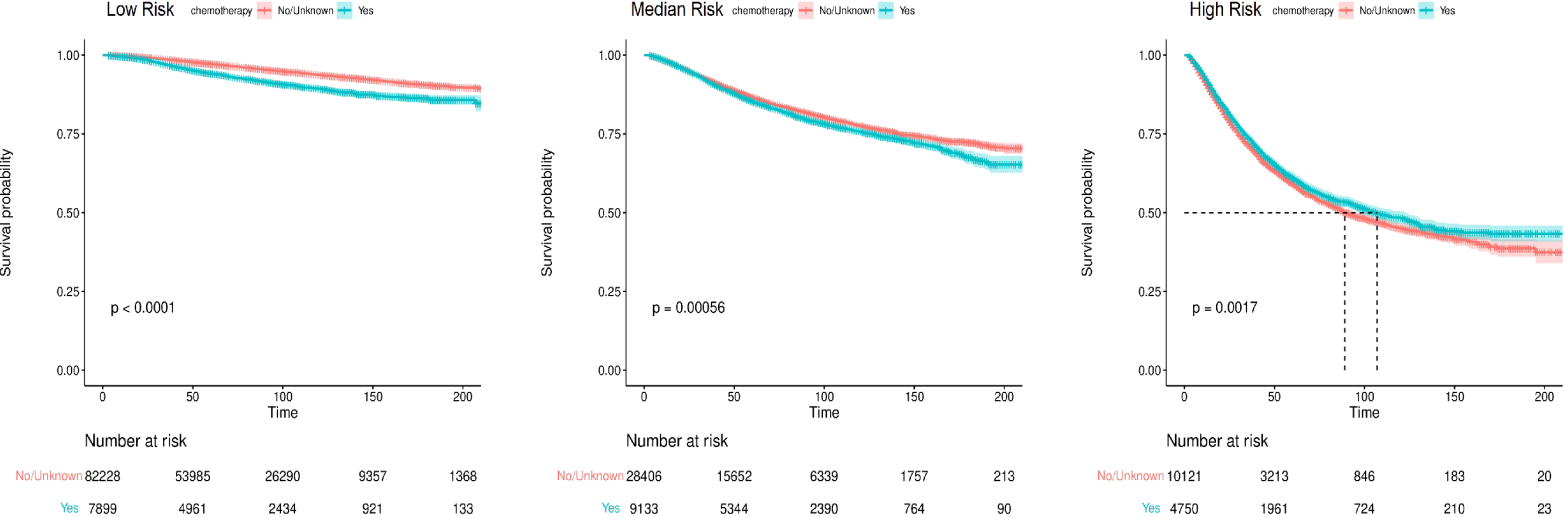
The Kaplan-Meier survival curves for breast cancer-specific survival (BCSS) according to risk stratification.

The nomogram-predicted total score was used for risk stratification by X-tile software (**Figure 7**), and the Kaplan–Meier curve was employed to show the survival difference of each risk group. As shown in **Figure 8**, patients in the high-risk group had better survival after receiving chemotherapy (*P*=0.0017). In the high-risk group, the 3-year, 5-year, and 10-year BCSS rates were 73.3%, 61.0%, and 48.3% for patients receiving chemotherapy, compared with 71.0%, 59.2%, and 45.0% for those who did not receive chemotherapy, respectively. However, for patients in the low-risk and median-risk groups, chemotherapy did not improve BCSS. In the low-risk group, the non-chemotherapy group had a better outcome (the 3-year, 5-year, and 10-year BCSS rates were 98.6%, 97.2%, and 93.7%) than the chemotherapy group (the 3-year, 5-year, and 10-year BCSS rates were 96.9%, 94.2%, and 89.4%) (*P*<0.0001). Similarly, chemotherapy was not recommended for patients in the median-risk group (*P*=0.00056), the 3-year, 5-year, and 10-year BCSS rates were 92.1%, 86.6%, and 77.6% for patients who did not receive chemotherapy compared with 91.4%, 85.2%, and 75.7% for those who received chemotherapy.

## 4 Discussion

Elderly women constitute an important part of breast cancer patients and have different biological and clinical characteristics compared with younger women. The physical decline of elderly patients increases with age, and poor physical status, many accompanying chronic diseases, and poor tolerance to treatment are also notable characteristics of such patients. In addition, due to the lack of prospective studies in elderly patients, the beneficial outcomes of chemotherapy in such patients are still controversial, and the actual practice is still influenced by the subjective opinion of clinicians, who lack reliable evidence to guide their treatment plans. In conclusion, it remains uncertain whether adjuvant chemotherapy translates into survival benefit after 70 years old. Therefore, it is of great significance to develop an individualized-level disease risk assessment model.

Sharon et al, in an observational study based on data collected from the SEER database, used Logistic regression analysis to determine factors related to chemotherapy and Cox proportional hazards models to calculate the hazard of death for patients with and without chemotherapy (10). Another study based on the SEER database used propensity score methods and multivariate proportional hazards regression to evaluate the effect of chemotherapy for patients with hormone receptor (HR)-negative breast cancer (11). However, the effects of other tumor variables, such as lymph node status, tumor size and grade, and HER2 expression, were not analyzed, and the subgroup of elderly women most likely to benefit from chemotherapy remains uncertain. In this study, we compared clinicopathological characteristics between the chemotherapy group and the non-chemotherapy group and performed PSM and IPTW matching analyses to ensure that difference in outcome was not due to demographic or pathological baseline imbalance between the two groups. The risk factors affecting BCSS were screened out by univariate and multivariate Cox regression analyses, and a predictive nomogram was constructed accordingly. It is verified that the model has good predictive performance. Our research can help clinicians accurately screen out patients who can benefit from chemotherapy and provide a reference for clinical treatment.

Whether elderly patients can truly benefit from chemotherapy is beyond doubt. Tamirisa et al. found that chemotherapy improved overall survival in elderly breast cancer patients with positive nodes, positive estrogen receptor and multiple comorbidities (12). The EBCTCG meta-analysis showed that patients over 70 years old have improved recurrence-free and overall survival after receiving combination chemotherapy, even though this benefit appeared to diminish with increasing age (13). Similarly, in the CALGB trial, 633 patients over 65 years old were randomized to receive cyclophosphamide, methotrexate, fluorouracil (CMF), doxorubicin and cyclophosphamide (AC), or capecitabine (14). The findings showed that patients who received single-agent chemotherapy had a doubled risk of relapse or death, suggesting the advantage of the combination therapy in this age group, even though the toxicity was pronounced. In a retrospective pooled analysis of 4 randomized trials, Muss et al. reported that chemotherapy reduced breast cancer mortality and recurrence rates with similar effects to younger women (6). Our findings suggested that patients in the high-risk group were with larger tumors, more positive lymph nodes, higher grade, HR-HER2- subtype and absence local therapy, who could obtain the improvement of BCSS after receiving chemotherapy. This conclusion is in accordance with the recommendation of the Society of Geriatric Oncology that older patients with node-positive, hormone-negative breast tumors may have the largest survival gain from chemotherapy (15).

Elderly breast cancer patients can benefit from chemotherapy but also suffer from inevitable chemotherapy toxicity. The general decline in physiological reserves and the increase in comorbidities predispose elderly women to a higher risk of toxicity. These include neuropathy, reduced left ventricular ejection fraction, congestive heart failure, myelodysplasia, acute leukemia, cardiotoxicity and secondary hematological malignancies (16–18). Another study reported that 24% of patients over 65 years old treated with docetaxel chemotherapy were hospitalized due to chemotherapy toxicity (19). In addition, anthracycline chemotherapy drugs have a higher risk of inducing myelosuppression in elderly breast cancer patients, with grade 3-4 myelosuppression occurring in 32% of patients, compared with 21% in younger patients. The difference between the two was statistically significant (*P*<0.0001) (20). Therefore, the chemotherapy of elderly women should be individualized, and chemotherapy toxicity must be carefully weighed. Especially for patients older than 80, our study implied these patients were classified into the high-risk group and more likely to benefit from chemotherapy. However, the toxic side effects of chemotherapy can lead to reduced quality of life in the extremely old patients. Therefore, it is necessary to make treatment decisions based on the geriatric assessment.

Currently, we often use different prognostic scores, such as Oncotype-DX, to determine the necessity of adjuvant chemotherapy. Oncotype-DX can assess the chemotherapy benefit and risk of recurrence within 10 years after breast cancer surgery in HR-positive, HER2-negative, node-negative, or limited to 1-3 node-positive patients. In addition, MammaPrint can also be used to assess the risk of recurrence, but its ability to predict chemotherapy response has not been proven. Our model aimed to predict the chemotherapy benefit at elderly patients regardless of HR, HER2 status, and lymph node metastasis, and none genetic sequencing technics was required to complete the evaluation, which showed a broad application scope especially in economically disadvantaged areas. Importantly, our model has higher clinical value for elderly patients who don’t meet the Oncotype-DX and Mammaprint detection indications. In the context of increased life expectancy, the treatment of elderly patients should be individualized to balance the benefits of chemotherapy and the loss of quality of life due to chemotherapy toxicity, and age should not be seen as a barrier to chemotherapy and management (21, 22). In contrast, we should comprehensively evaluate the patient’s general condition, cardiopulmonary and other organ functions, complications, and social support. Poor physical condition, more complications, etc., can lead to increased all-cause mortality, making adjuvant chemotherapy redundant. In conclusion, a comprehensive geriatric assessment (CGA) plays an important role in treatment decisions for elderly patients.

Although the TNM staging system is an important tool for predicting prognosis, some important prognostic factors, such as age, were not included, and the accuracy of the system’s predictive results was insufficient. Our nomogram not only contains the parameters of the AJCC staging system but also includes some individual demographic and pathological characteristics and can help doctors distinguish the benefit group from chemotherapy. Therefore, it provides more comprehensiveness and convenience. In addition, elderly breast cancer patients often die from chronic diseases such as heart disease, lung disease, and cerebrovascular disease, not from breast cancer itself (23). Traditional survival analysis methods such as Kaplan-Meier analysis and Cox proportional hazards regression analysis, which treat deaths from other causes as censored events, tend to overestimate breast cancer mortality (24). Therefore, BCSS was chosen as the endpoint in this study, which excludes the effect of death from other diseases, making the results more accurate. However, our study also has certain limitations. First, due to limited database permissions, the information related to chemotherapy and radiation was only in the categories of “Yes” and “No/unknown”. There was no information about chemotherapeutic agents, doses, number of cycles, and toxicity. Second, the SEER database also did not provide some important prognostic-related features, including HER-2 status before 2010, and tumor progression, which affected the validity of our model. Moreover, since there is no Ki67 record in SEER database, we divided breast cancer molecular types according to HR and HER2 status as follows: HR+HER2+, HR-HER2+, HR+HER2-, HR-HER2-. A limitation of this classification is that HR+HER2- includes Luminal A and Luminal B, which affects the application of the model. In addition, characteristics of geriatric assessment, such as comorbidities, physical functional status, mental health, and social support, were not included in the model. Finally, this is a retrospective study with unavoidable selection bias. To enhance the convincing power of the model, the nomogram should pass further prospective research for confirmation and supplementation.

In conclusion, our study suggests that women older than 70 years with larger tumors, higher grade, positive nodes, negative hormone receptor and inactive local therapy who are relatively healthy should receive chemotherapy. In addition, the benefit of chemotherapy and the loss of quality of life due to chemotherapy toxicity should be assessed individually. Our findings also support the view that conventional chemotherapy should be administered cautiously to older women with a favorable prognosis in the low- and median-risk group.

## Data availability satement

The original contributions presented in the study are included in the article/**Supplementary Material**. Further inquiries can be directed to the corresponding authors.

## Author contributions

SP: Conceptualization, Methodology, Validation, Writing - Original Draft. PX: Formal analysis, Investigation. HC: Software, Visualization. YL: Data Curation, Resources. JH: Writing- Reviewing and Editing, Supervision. HZ: Conceptualization, Writing - Review and Editing. All authors read and approved the final manuscript.
