# Supplementary figures and images for: Evaluation of outcome of chemotherapy for breast cancer patients older than 70 years: A SEER-based study
Source: Front Oncol. 2023 Mar 28;13:992573. doi: 10.3389/fonc.2023.992573 (PMC10086342; doi:10.3389/fonc.2023.992573)

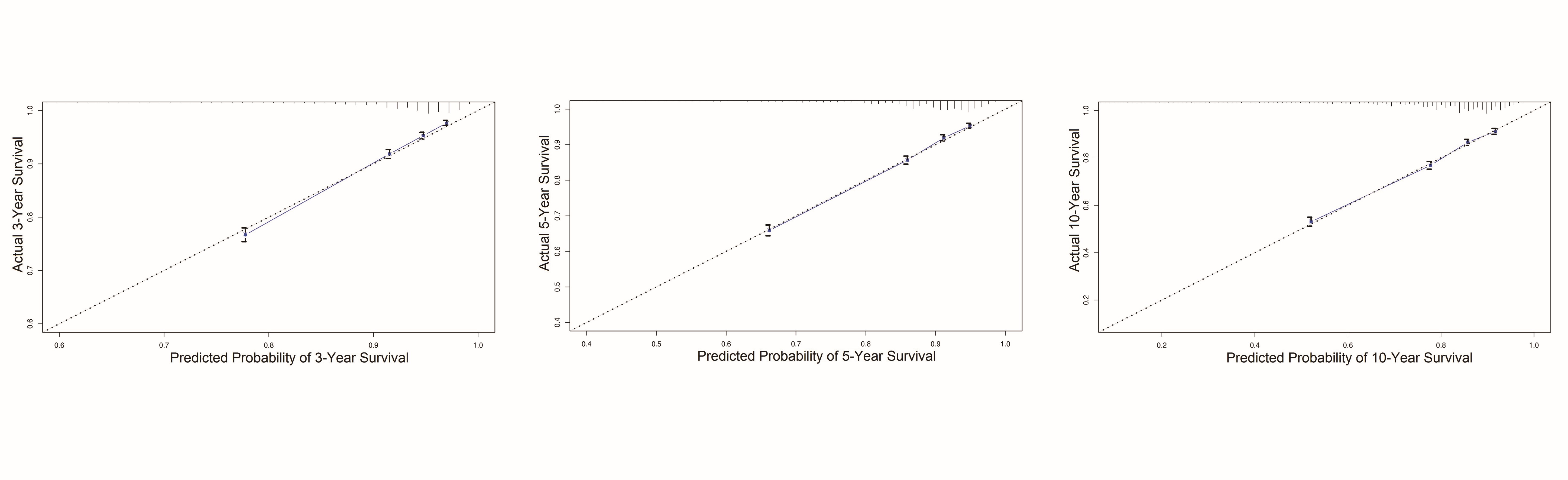

Supplement: Supplementary file 1 [file Image_1.jpeg]
