# Supplementary material for: Evaluation of outcome of chemotherapy for breast cancer patients older than 70 years: A SEER-based study
Source: Front Oncol. 2023 Mar 28;13:992573. doi: 10.3389/fonc.2023.992573 (PMC10086342; doi:10.3389/fonc.2023.992573)
Supplement: Supplementary file 2 [file Table_1.docx]

Supplementary Table

Supplementary table 1: The clinicopathological characteristics of the unmatched and propensity score matched (PSM) patients.

|  | **Unmatched** | |  | **Propensity Matched** | |  |
| --- | --- | --- | --- | --- | --- | --- |
| **Variables** | **No/Unknown** | **Yes** |  | **No/Unknown** | **Yes** |  |
|  | **N=120755 (%)** | **N=21782 (%)** | ***P*-value** | **N=18413 (%)** | **N=18413 (%)** | ***SMD*** |
| **Age** |  |  |  |  |  |  |
| 70-77 | 60443 (50.1) | 17019 (78.1) | 0.000 | 13498 (73.3) | 13922 (75.6) | 0.054 |
| 78-84 | 40587 (33.6) | 4077 (18.7) |  | 4137 (22.5) | 3814 (20.7) |  |
| >=85 | 19725 (16.3) | 686 (3.15) |  | 778 (4.2) | 677 (3.7) |  |
| **Race** |  |  |  |  |  |  |
| Black | 8342 (6.91) | 2333 (10.7) | 0.000 | 1916 (10.4) | 1810 (9.8) | 0.027 |
| Other | 7153 (5.92) | 1411 (6.48) |  | 1169 (6.3) | 1264 (6.9) |  |
| White | 105260 (87.2) | 18038 (82.8) |  | 15328 (83.2) | 15339 (83.3) |  |
| **Marital** |  |  |  |  |  |  |
| No | 71242 (59.0) | 11067 (50.8) | 0.000 | 9841 (53.4) | 9484 (51.5) | 0.039 |
| Yes | 49513 (41.0) | 10715 (49.2) |  | 8572 (46.6) | 8929 (48.5) |  |
| **Laterality** |  |  |  |  |  |  |
| Left | 61207 (50.7) | 11297 (51.9) | 0.001 | 9544 (51.8) | 9547 (51.8) | 0.001 |
| Right | 59548 (49.3) | 10485 (48.1) |  | 8869 (48.2) | 8866 (48.2) |  |
| **Grade** |  |  |  |  |  |  |
| I | 32655 (27.0) | 1577 (7.24) | 0.000 | 1381 (7.5) | 1556 (8.5) | 0.049 |
| II | 57871 (47.9) | 7393 (33.9) |  | 6642 (36.1) | 6862 (37.3) |  |
| III | 29494 (24.4) | 12576 (57.7) |  | 10181 (55.3) | 9802 (53.2) |  |
| IV | 735 (0.61) | 236 (1.08) |  | 209 (1.1) | 193 (1.0) |  |
| **Stage** |  |  |  |  |  |  |
| I | 78634 (65.1) | 5328 (24.5) | 0.000 | 4952 (26.9) | 5276 (28.7) | 0.058 |
| II | 34917 (28.9) | 10586 (48.6) |  | 9690 (52.6) | 9162 (49.8) |  |
| III | 7204 (5.97) | 5868 (26.9) |  | 3771 (20.5) | 3975 (21.6) |  |
| **Stage_T** |  |  |  |  |  |  |
| T1 | 88328 (73.1) | 9572 (43.9) | 0.000 | 8660 (47.0) | 8867 (48.2) | 0.033 |
| T2 | 27328 (22.6) | 9346 (42.9) |  | 7825 (42.5) | 7532 (40.9) |  |
| T3 | 2607 (2.16) | 1362 (6.25) |  | 935 (5.1) | 988 (5.4) |  |
| T4 | 2492 (2.06) | 1502 (6.90) |  | 993 (5.4) | 1026 (5.6) |  |
| **Stage_N** |  |  |  |  |  |  |
| N0 | 98727 (81.8) | 9663 (44.4) | 0.000 | 8950 (48.6) | 9108 (49.5) | 0.045 |
| N1 | 17296 (14.3) | 7584 (34.8) |  | 6631 (36.0) | 6269 (34.0) |  |
| N2 | 3256 (2.70) | 2907 (13.3) |  | 1899 (10.3) | 2051 (11.1) |  |
| N3 | 1476 (1.22) | 1628 (7.47) |  | 933 (5.1) | 985 (5.3) |  |
| **Local_therapy** |  |  |  |  |  |  |
| BCS | 26206 (21.7) | 2569 (11.8) | 0.000 | 2436 (13.2) | 2308 (12.5) | 0.082 |
| BCS+Radiation | 46644 (38.6) | 7629 (35.0) |  | 6391 (34.7) | 6895 (37.4) |  |
| Mastectomy | 40675 (33.7) | 7108 (32.6) |  | 6937 (37.7) | 6349 (34.5) |  |
| Mastectomy+Radiation | 3414 (2.83) | 3737 (17.2) |  | 2047 (11.1) | 2282 (12.4) |  |
| Radiation | 32 (0.03) | 31 (0.14) |  | 19 (0.1) | 19 (0.1) |  |
| No | 3784 (3.13) | 708 (3.25) |  | 583 (3.2) | 560 (3.0) |  |
| **Subtype** |  |  |  |  |  |  |
| HR-/HER2- | 3399 (2.81) | 2542 (11.7) | 0.000 | 1786 (9.7) | 1720 (9.3) | 0.021 |
| HR-/HER2+ | 957 (0.79) | 1114 (5.11) |  | 622 (3.4) | 635 (3.4) |  |
| HR+HER2- | 40380 (33.4) | 4194 (19.3) |  | 3792 (20.6) | 3876 (21.1) |  |
| HR+/HER2+ | 2717 (2.25) | 2103 (9.65) |  | 1463 (7.9) | 1391 (7.6) |  |
| Not 2010+ | 73302 (60.7) | 11829 (54.3) |  | 10750 (58.4) | 10791 (58.6) |  |

Notes: BCS: Breast conserving surgery; HR: Hormone receptor; HER2: Human epidermal growth factor receptor 2; PSM: propensity score matched; SMD: Standardized mean differences.

Supplementary table 2. Univariate and multivariable analysis of breast cancer-specific survival predictors in patients after propensity score matching (PSM).

|  | **Univariate analysis** | | | **Multivariate analysis** | | |
| --- | --- | --- | --- | --- | --- | --- |
| **Variables** | **HR*** | **95%CI** | ***P-*value** | **HR*** | **95%CI** | ***P-*value** |
| **Age** |  |  |  |  |  |  |
| >=85 | Reference |  |  | Reference |  |  |
| 70-77 | 0.242 | (0.206-0.285) | 0.000 | 0.552 | (0.464-0.657) | 0.000 |
| 78-84 | 0.489 | (0.413-0.579) | 0.000 | 0.749 | (0.629-0.892) | 0.001 |
| **Race** |  |  |  |  |  |  |
| Black | Reference |  |  | Reference |  |  |
| Other | 0.620 | (0.502-0.765) | 0.000 | 0.738 | (0.597-0.913) | 0.005 |
| White | 0.667 | (0.590-0.754) | 0.000 | 0.832 | (0.734-0.943) | 0.004 |
| **Laterality** |  |  |  |  |  |  |
| Left | Reference |  |  |  |  |  |
| Right | 0.929 | (0.855-1.009) | 0.082 |  |  |  |
| **Marital** |  |  |  |  |  |  |
| No | Reference |  |  | Reference |  |  |
| Yes | 0.670 | (0.616-0.730) | 0.000 | 0.906 | (0.830-0.989) | 0.027 |
| **Grade** |  |  |  |  |  |  |
| I | Reference |  |  | Reference |  |  |
| II | 2.440 | (1.837-3.24) | 0.000 | 1.732 | (1.303-2.303) | 0.000 |
| III | 4.799 | (3.64-6.327) | 0.000 | 2.867 | (2.168-3.789) | 0.000 |
| IV | 6.007 | (4.097-8.806) | 0.000 | 3.860 | (2.628-5.670) | 0.000 |
| **T stage** |  |  |  |  |  |  |
| T1 | Reference |  |  | Reference |  |  |
| T2 | 3.026 | (2.732-3.353) | 0.000 | 2.119 | (1.904-2.358) | 0.000 |
| T3 | 5.734 | (4.900-6.711) | 0.000 | 2.547 | (2.152-3.015) | 0.000 |
| T4 | 7.993 | (6.918-9.236) | 0.000 | 3.278 | (2.791-3.850) | 0.000 |
| **N stage** |  |  |  |  |  |  |
| N0 | Reference |  |  | Reference |  |  |
| N1 | 1.768 | (1.593-1.962) | 0.000 | 1.727 | (1.550-1.923) | 0.000 |
| N2 | 4.317 | (3.829-4.868) | 0.000 | 3.168 | (2.781-3.609) | 0.000 |
| N3 | 7.448 | (6.506-8.525) | 0.000 | 4.366 | (3.776-5.048) | 0.000 |
| **Local_therapy** |  |  |  |  |  |  |
| BCS | Reference |  |  | Reference |  |  |
| BCS+Radiation | 0.483 | (0.415-0.562) | 0.000 | 0.627 | (0.538-0.731) | 0.000 |
| Mastectomy | 1.274 | (1.116-1.455) | 0.000 | 0.994 | (0.869-1.138) | 0.935 |
| Mastectomy+Radiation | 1.726 | (1.478-2.015) | 0.000 | 0.801 | (0.679-0.944) | 0.008 |
| Radiation | 1.681 | (0.539-5.244) | 0.371 | 0.931 | (0.297-2.922) | 0.902 |
| No | 5.360 | (4.414-6.508) | 0.000 | 3.412 | (2.775-4.195) | 0.000 |
| **Subtype** |  |  |  |  |  |  |
| HR-/HER2- | Reference |  |  | Reference |  |  |
| HR-/HER2+ | 0.974 | (0.747-1.270) | 0.846 | 0.840 | (0.644-1.096) | 0.199 |
| HR+/HER2- | 0.423 | (0.351-0.509) | 0.000 | 0.421 | (0.347-0.510) | 0.000 |
| HR+/HER2+ | 0.641 | (0.514-0.799) | 0.000 | 0.620 | (0.496-0.774) | 0.000 |
| Not 2010+ | 0.602 | (0.521-0.695) | 0.000 | 0.594 | (0.511-0.690) | 0.000 |

Notes: BCS: Breast conserving surgery; HR: Hormone receptor; HER2: Human epidermal growth factor receptor 2; HR*: hazard ratio; CI: confidence interval.
